# The New Year, 1918: What to Read and Study

**Published:** 1917-12-29

**Authors:** 


### THE MATRONS' AND SISTERS' DEPARTMENT

### THE NEW YEAR, 1918.

### WHAT TO READ AND STUDY.

THE experiences of a busy life, as age presses upon men and women, may provide supreme Consolation and Hope. Consolation, in the knowledge which comes to the observant, that, those who believe and rest their inner life upon Christ's teaching and work, do attain to a peace of mind, which passes all understanding. Hope, that in God's good providence the opportunities life affords, by intercourse with one's fellows, and the confidences and counsel sought and exchanged, may be utilised to steady the anxious, and to build up the courage and strengthen the resolution of the faint-hearted and doubting. Oh! the privilege and joy of this closer intimacy with our fellow-workers, and the added strength and peace it can bring to all.

Fifty years' association with the sick, and those who spend a measure of their lives in ministering to their needs, has enlarged the heart and illuminated the understanding. Here in the Homeland how many, alas, have still to shed the old habits of thought and narrowness. But to lives spent in tent and hut hospitals at the Front, where patients flow in continuously from the battlefields, where Death makes his presence felt with poignant persistence, the atmosphere becomes so continuously charged with apprehension of the living reality of the spiritual nature in each man and woman, that, it is, in fact, the most inspiriting force they can or do feel on this side the grave. Working under such conditions makes for glorious changes in many things. There is no need for the continuous oversight and force of a controlling hand. For all, from the humblest worker to the highest, are inspired with the will to do always their utmost and their best, as if each day was the last occasion open to each man and woman to render personal service.

This terrible war is coming ever closer and nearer to all left at home. May we not hope that with the New Year the atmosphere of our battlefield hospitals may continuously be that of every hospital worker on this side the Channel? Such a change would make their spiritual natures a living force, quickened with new hope, making the atmosphere of our hospitals everywhere identical, and full of fresh life and high purpose. May we enter on the New Year inspired with this hope, uniting the writer and reader in a purposeful endeavour to hasten its fulfilment?

What may helpfully be the special reading of all workers during 1918? The taking of Jerusalem by our Forces, its ransom by the Armies of a Christian Nation, which by proclamation has ensured protection for the shrines of all the faiths throughout the Holy Land, have fired the imagination of the peoples of the world. Once again Christ reigns at Bethlehem, to which all devout believers' hearts went out with renewed and quickened faith on Christmas Day. Christ's teachings and their study have so become the active pursuit of innumerable persons, and of these not a few are able workers who properly regard personal service as a quickening force to their spiritual natures. And amongst books there is no single volume to compare with the Bible. It is the book which never palls, for the more often it is read, and the wider the life experiences of the reader, the fresher and more wonderful are the truths which reveal themselves in its pages. Once the daily reading of the Bible has been acquired, it can never be omitted without a feeling of regret and loss. So true is it that within its pages is to be found the compass, which will enable the reader to pursue life's journey with wisdom and increasing delights. The inspiration, assured knowledge, and unfailing guidance thus derived, quicken our spiritual nature, and assure us of the reality of the presence of Almighty God, and the sufficiency of Christ's teaching and example.

Here is the experience, not of one but of all members of the human race who have been drawn to acquire the daily study of the Bible. We know no special reading of equal fruitfulness and hope, that can be found in this world. The direction of the thoughts and imagination of men and women to Jerusalem, and all its history and memories mean to the nation, may well cause the individual to turn to the Bible with hope, and to read and study it habitually. We wish every reader may be moved to adopt this course throughout 1918.

